# Packaging impact on quality changes of dried Liang leaf seasoning

**DOI:** 10.1016/j.heliyon.2024.e40462

**Published:** 2024-11-15

**Authors:** Worapong Usawakesmanee, Supachai Pisuchpen, Sunisa Siripongvutikorn, Nicha Khatcharin, Chanonkarn Rujirapong

**Affiliations:** a Centre of Excellence in Functional Foods and Gastronomy, Faculty of Agro-Industry Prince of Songkla University, Hat Yai, Songkhla, 90110, Thailand; b Centre of Excellence in Bio-based Materials and Packaging Innovation, Faculty of Agro-Industry Prince of Songkla University, Hat Yai, Songkhla, 90110, Thailand

**Keywords:** Antioxidant, Bioactive, Rice seasoning, Food supplement, *Gnetum gnemon*, Vegetable, Shelf life

## Abstract

This study aimed to develop and characterize a dried seasoning made from Liang (*Gnetum gnemon* var. *tenerum*) leaves, with a focus on the effects of packaging and storage conditions on the quality and shelf life. The seasoning, rich in protein (25.20 g/100 g), fiber (10.22 g/100 g), vitamin B_2_ (0.52 mg/100 g), and calcium (642.17 mg/100 g), was stored in laminated aluminum foil and nylon/LLDPE packaging at 30 °C with 60 % and 90 % relative humidity (RH) for 12 weeks. Laminated aluminum foil exhibited superior moisture barrier properties, with a water vapor transmission rate (WVTR) of 3.22 × 10⁻⁶ g/(day × cm^2^) at 25 °C and RH 50 % and a water vapor permeability coefficient (P) of 2.52 × 10^⁻5^ g × μm/(day × cm^2^ × mmHg), making it 19 times more effective as a barrier material compared to nylon/LLDPE. As a result, the seasoning stored in laminated aluminum foil at RH 60 % maintained the lowest moisture content (3.45–3.58 %) and water activity (0.277–0.278) and demonstrated the longest estimated shelf life of 684 days, compared to only 12 days for nylon/LLDPE at RH 90 %. Antioxidant activity, total phenolic content (TPC) and total flavonoid content (TFC) remained relatively stable over the 12-week storage period, with at least 70 % of their initial levels. During storage, the number of microbes appeared to increase, yet remained below 3 log CFU/g for the total viable count and 2 log CFU/g for yeast and mold. The seasoning packaged in laminated aluminum foil also yielded the highest sensory scores. Overall, laminated aluminum foil at RH 60 % proved to be the most effective packaging material for extending the shelf life and maintaining the quality of dried Liang leaf seasoning.

## Highlights

• Novel Dried Seasoning as Food Supplement: shelf-stable Liang leaf seasoning rich in protein, fiber, vitamins, minerals, and antioxidants.
• Preserved Bioactives: Total phenolics remained stable, whereas flavonoids and antioxidants initially decreased, then stabilized during storage.
• Extended Shelf Life: Laminated aluminum foil packaging preserved quality, extending shelf life to 684 days at RH 60%.

## Graphical abstract

**Figure:**
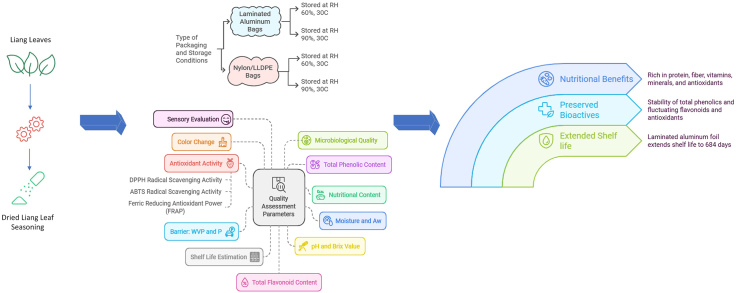

## 1 Introduction

A well-balanced diet, rich in fruits and vegetables, is essential for promoting and maintaining good health. These plant-based foods provide an extensive array of vital nutrients, including dietary fiber, vitamins, minerals, and phytochemicals. The consistent consumption of fruits and vegetables offers substantial health benefits, such as antioxidant and anti-inflammatory effects, which contributes to the prevention of various diseases [1]. Despite these advantages, inadequate consumption of fruits and vegetables remains a significant global health issue, contributing to approximately 3 % of the deaths worldwide [2,3]. Several factors contribute to this shortfall include personal preferences, limited market availability, unappealing sensory characteristics, concerns regarding pesticide use, and financial constraints [2,4].

Fresh produce is highly perishable and prone to spoilage during harvesting, transport, and storage due to its high-water content, respiratory rate, delicate structure, and susceptibility to microbial contamination [5]. To address this issue, drying has long been used as a simple and effective preservation method by removing excess moisture through heat and mass transfer; drying inhibits microbial growth and extends shelf life of food. However, dried foods can undergo chemical and physical changes such as oxidation, enzymatic reactions, and fracturing [6].

In Southern Thailand*, Gnetum gnemon* var. *tenerum*, commonly known as Liang, is a prevalent leafy green vegetable. Consumed fresh, stir-fried, or boiled, Liang has become a local culinary favorite, often recommended to guests as a must-try dish. Liang leaves are distinguished from other leafy greens because of their unique, mild flavor and nutritional profile, particularly their high protein content, and complete amino acid profile, which are uncommon in leafy vegetables [7,8]. Beyond its pleasant taste and texture, Liang leaves offer potential health benefits. Anisong et al. [9] found that undigested Liang leaves contained GABA, an important neurotransmitter, in a gut model system that was not found in green tea samples. Additionally, Suksanga et al. [10] found that Liang powder improved the internal organs of induced diabetic Wistar rats. The antioxidant, antidiabetic, and anti-inflammatory properties of Liang leaves are attributed to their rich contents of phenolic compounds, flavonoids, fiber, and amino acids [7,8]. The health benefits of Liang leaves have caught the attention of the food industry, with a prominent corporation recently introducing ready-to-eat Liang meals in convenience stores to meet the increasing consumer demand. Furthermore, Liang leaves show promise as an alternative ingredient to furikake, a worldwide Japanese rice seasoning [11]. Traditional furikake often contains dried seaweed [11], which has raised health concerns due to potential heavy metal contamination from marine pollution. Liang leaves could offer similar sensory benefits without these associated risks [12].

Continuing from previous study, this study aimed to develop and characterize the impact of packaging on dried Liang leaf seasoning. Dried seasoning was developed and prepared from indigenous Liang leaves following the preparation method of Siripongvutikorn et al. [12], and changes in quality during storage under various packaging and storage conditions were evaluated. This study seeks to provide comprehensive data to support future manufacturing and marketing efforts, contribute to the diversification of healthy food products, and promote sustainable use of local plant resources.

## 2 Materials and methods

This research involving human sensory activities was approved by the PSU Human Research Ethics Committee (reference No. 2023-005-1-1).

### 2.1 Production of dried seasoning from Liang leaves

**Fig. 1:**
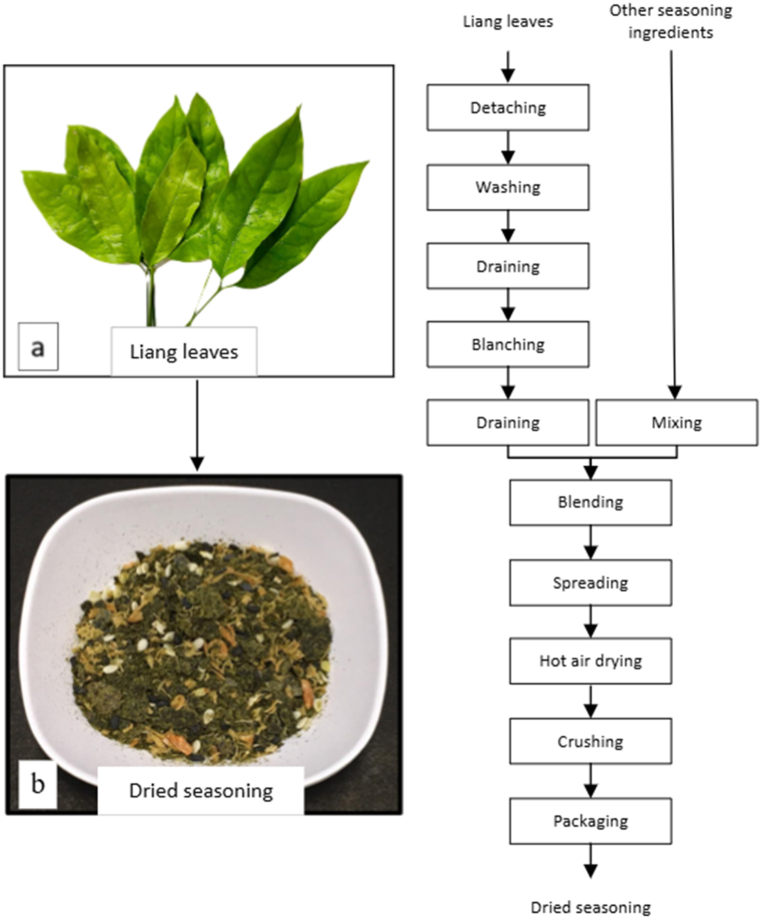
Preparation process of dried seasoning.

A flowchart of the dried seasoning preparation process is presented in Fig. 1. Liang leaves in the Pae Salat state (Fig. 1(a)) were sourced from a local agricultural supplier for preparation of a dried seasoning. The preparation process was initiated by separating leaves from stems. To ensure adequate sanitation, leaves were soaked in water containing 100 ppm chlorine for 15 min. After this treatment, the leaves were rinsed twice with flowing tap water to remove residual chlorine. After draining for 10 min in a basket with controlled overlay thickness not exceeding 1 cm, the leaves were blanched in hot water containing 1 % NaCl for 3 min and immediately cooled in cold water, with excess water drained. The blanched leaves were then ground into a paste and mixed thoroughly with dried shrimp and seasoning ingredients, such as garlic powder, black sesame, white sesame, and soy sauce, in undeclared amounts due to petty patent and entrepreneur requirements. The well-blended paste was spread evenly and dried in a hot air oven at 70 °C for 5 h. After drying, the mixture was crushed into coarse granules (Fig. 1(b)) and packaged into 15 cm × 10.5 cm laminated aluminum foil (93.0 μm thick) and nylon/LLDPE bags (77.6 μm thick) for storage testing. Packaged samples (4 g) were stored at 30 °C, relative humidity (RH) 60 %, and 90 % for 12 W to evaluate the quality changes during storage. Four treatments included laminated aluminum bags stored at RH 60 % (A60) and, RH 90 % (A90), and nylon/LLDPE bags stored at RH 60 % (N60) and RH 90 % (N90).

### 2.2 Nutrition content

The nutritional composition of the products was analyzed both initially and after a 12-week storage period. This analysis was conducted by the ISO/IEC 17025:2017 accredited Centre of Measurement and Standard at the Faculty of Science, Prince of Songkla University. The assessment included measurements of moisture, protein, fat, ash, fiber, sugars, saturated fatty acids, cholesterol, and specific minerals (sodium, calcium, and iron), all of which were quantified following the standardized protocols established by the Association of Official Analytical Chemists [13]. Carbohydrate content was analyzed according to the methodology outlined by Ellefson [14]. The determination of vitamin A, B1, and B2 was conducted using methods outlined by Visuthi [15], Woollard and Indyk [16], and Wehling and Wetzel [17], respectively. The energy value was assessed according to the protocol established by Sullivan [18].

### 2.3 Moisture and a_w_

Moisture content and water activity (a_w_) of the products were analyzed using the oven method [19] and an a_w_ analyzer (Aqualab Pre, Decagon Devices Inc., Washington, USA), respectively.

### 2.4 Water vapor transmission rate and permeability coefficient

The water vapor transmission rate (WVTR) of the films was measured using the ASTM E96-80 standard cup gravimetric method. Circular samples with a diameter of 7 cm were cut from each of the triplicate specimens and placed over aluminum cups containing anhydrous silica gel. Each sample was sealed with a silicone ring and stored in a humidity-controlled chamber at 25 °C and RH 50 %. The cups were weighed daily using an analytical balance over a period of 7 days. The WVTR value was obtained by calculating the slope of the graph by plotting the storage time against the weight gain of the cup and considering the exposed surface area of the films. The permeability coefficient (P) was calculated using Equation (1).

(1) $$P=\frac{WVTR\times l}{\Delta p}$$

where: WVTR = water vapor transmission rate,

l = film thickness, and.

Δp = water vapor pressure difference across the film.

### 2.5 Moisture content change

Changes in moisture content were assessed by measuring moisture uptake during storage under two controlled environmental conditions: 30 °C with RH 60 % and 30 °C with RH 90 %. The samples were weighed biweekly over a 12-week period and stored in their respective packaging materials. At each measurement interval, the moisture content of the packaged products was calculated using Equation (2).

(2) $$\text{Mt}=\left[\frac{Wt\times \left(1+Mi\right)}{Wi}-1\right]$$

where: Mt = product moisture content at time t,

Wi = initial weight of sample, and.

Wt = weight of sample at time t.

### 2.6 Shelf life estimation of dried seasoning

The shelf life of the dried seasoning was determined based on water vapor permeation through the packaging film and the storage conditions. Moisture content has emerged as a critical factor influencing seasoning stability and is considered the primary indicator for shelf life estimation. Sample was subjected to a controlled environment of 30 °C with RH 75 %. Sensory crispness was monitored hourly until it reached an unacceptable level. The critical moisture content and water activity of the sample were analyzed. The shelf life of 4 g of dried seasoning in a 15 cm × 10.5 cm package was then calculated using Equation (3).

(3) $$t=\frac{q\times l}{A\times P\times \Delta p}$$

where: q = quantity of absorbed moisture by the product to reach the critical moisture content,

l = film thickness,

A = film area (315 cm^2^),

P = permeability coefficient of the film,

Δp = water vapor pressure difference across the film

4a $$\Delta p=\frac{1}{2}\times \text{ps}\times \left(\left(a_{w}o‐a_{w}i\right)+\left(a_{w}o‐a_{w}c\right)\right)$$

ps = saturated water vapor pressure at storage temperature (31.824 mmHg),

*a*_w_o = water activity of storage conditions (0.6 for RH 60 % and 0.9 for RH 90 %),

*a*_w_i = initial water activity of samples (0.22), and

*a*_w_c = critical water activity of samples (0.57).

### 2.7 pH and Brix value

pH and Brix values were measured using a pH meter (Docu-pH + Meter, Sartorius AG, Göttingen, Germany) and a refractometer (Pen refractometer, Atago, Tokyo, Japan), respectively.

### 2.8 Color change

The CIE L∗, a∗, and b∗ systems were employed to evaluate color changes using a colorimeter (ColorFlex EZ, Hunter Associates Laboratory Inc., Virginia, USA). The results were reported by comparison to a freshly produced sample (standard) as ΔE, calculated using Equation (4).

4b $$\Delta E=\sqrt{{\left(\Delta L\right)}^{2}{+\left(\Delta a\right)}^{2}{+\left(\Delta b\right)}^{2}}$$

where: ΔE = color difference between standard,

ΔL = difference between lightness (L∗) standard,

Δa = difference between redness-greenness (a∗) standard, and.

Δb = difference between yellowness-blueness (b∗) standard.

### 2.9 Total phenolic content, total flavonoid content, and antioxidant activity

Total phenolic content (TPC), total flavonoid content (TFC), and antioxidant activity were quantified and expressed as standard equivalents per sample unit [20]. To evaluate the changes in these parameters over the storage period, the values obtained for the stored samples were compared with those of the freshly produced product using Equation (5).

$$\text{Relative}\;\text{value}\;\left(\%\right)=\frac{V\text{alue}}{I\text{nitial}}\times 100$$

where: Relative value = percentage comparative values,

Value = value of the product at a specific storage time, and.

Initial = value of the freshly produced product.

### 2.10 Sample preparation and extraction

A 1:10 (w/v) ratio of the sample to 90 % ethanol was prepared and agitated vigorously in the dark at 25 °C for 24 h. The resulting mixture was subsequently vacuum filtered through a Buchner funnel and centrifuged at 17,700×*g* for 15 min at 4 °C. The ethanol was then removed using an evaporator to concentrate the sample.

### 2.11 Total phenolic content

The total phenolic content (TPC) in the samples was quantified using a modified version of the method of Singleton and Rossi [21]. For this procedure, 20 μl of the sample extract was mixed with 100 μl of 10 % (v/v) Folin reagent in a 96-well plate. The mixture was incubated in the dark at 30 °C for 6 min, after which 7.5 % (w/v) sodium carbonate (Na₂CO₃) was added. The solution was incubated for an additional 30-min incubation. Absorbance was measured at 765 nm. Calibration curves were generated using three standard compounds: gallic acid (0–100 μg/ml, R^2^ = 0.999), Trolox (0–500 μg/ml, R^2^ = 0.999), and L-ascorbic acid (0–200 μg/ml, R^2^ = 0.999).

### 2.12 Total flavonoid content

Total flavonoid content was evaluated using a modified version of the method described by Ha et al. [22]. Equal volumes (100 μl each) of the sample extract and 2 % aluminum chloride (AlCl₃) solution (w/v) were combined. The resulting mixture was incubated in the dark at 30 °C for 60 min to ensure the optimal reaction conditions. After incubation, absorbance was measured at 420 nm. Quercetin and rutin were used as calibration standards with concentration ranges of 0–20 μg/ml (R^2^ = 0.999) and 0–80 μg/ml (R^2^ = 0.998), respectively.

### 2.13 DPPH radical scavenging activity

2,2-diphenyl-1-picryl hydrazyl (DPPH) radical scavenging activity was evaluated using a modified method based on the method of Brand-Williams et al. [23]. The procedure involved mixing equal volumes (100 μl each) of the sample extract and a 0.2 mM DPPH solution in 95 % ethanol. The mixture was incubated in the dark at 30 °C for 30 min. Following incubation, the absorbance was measured at 517 nm. Standard curves were prepared using gallic acid (0–2.5 μg/ml, R^2^ = 0.998), Trolox (0–12 μg/ml, R^2^ = 0.998), and L-ascorbic acid (0–14 μg/ml, R^2^ = 0.997) as reference standards.

### 2.14 ABTS radical scavenging activity

2,2-azino-bis-3-ethylbenzthiazoline-6-sulfonic acid (ABTS) assay was conducted following the protocol established by Arnao et al. [24]. ABTS radicals were generated by incubating a 7.4 mM ABTS solution in the dark at 30 °C for 12 h. This radical solution was then diluted to achieve an absorbance of 1.1±0.02 at 734 nm. To conduct the assay, a mixture of 20 μl of sample extract and 280 μl of radical solution was prepared and incubated in the dark at 30 °C for 2 h. The absorbance of the mixture was measured at 734 nm. Gallic acid, Trolox, and L-ascorbic acid served as standards with concentration ranges of 0–21 μg/ml (R^2^ = 0.998), 0–110 μg/ml (R^2^ = 0.999), and 0–110 μg/ml (R^2^ = 0.999), respectively.

### 2.15 Ferric reducing antioxidant power (FRAP)

The ferric reducing antioxidant power (FRAP) assay was conducted following the protocol established by Benzie and Strain [25]. FRAP reagent was prepared by mixing 300 mM acetate buffer (pH 3.6), 10 mM TPTZ (2,4,6-tri(2-pyridyl)-s-triazine in 40 mM HCl, and 20 mM FeCl_3_·6H2O in a 10:1:1 ratio. This solution was heated at 37 °C for 30 min to ensure complete reaction. Subsequently, 15 μl of the sample extract was added to 285 μl of the FRAP reagent, and the mixture was incubated at 37 °C for 30 min. Absorbance was measured at 593 nm to determine antioxidant activity. Gallic acid, Trolox, L-ascorbic acid, and FeSO_4_ were used as standards, with concentrations ranging from 0 to 12 μg/ml (R^2^ = 0.999), 0–100 μg/ml (R^2^ = 0.999), 0–100 μg/ml (R^2^ = 0.999), and 0–90 μg/ml (R^2^ = 0.999), respectively.

### 2.16 Microbiological quality

Microbial quality assessment of the dried seasoning was conducted by the Centre of Measurement and Standard Accreditation, which holds the ISO/IEC 17025:2017 accreditation. This evaluation covered several critical parameters, including total viable count (TVC), coliform bacteria, *Escherichia coli*, and yeast and mold (YM).

### 2.17 Sensory evaluation

A panel of 50 untrained evaluators assessed eight sensory attributes of the samples using a 9-point hedonic scale. The evaluated attributes included appearance, color, odor, texture, flavor, taste, overall acceptability, and consumer acceptance (percentage of consumer approval). This sensory evaluation was conducted in accordance with the ethical standards established by the PSU Human Research Ethics Committee (reference No. 2023-005-1-1).

### 2.18 Statistical analysis

A completely randomized design (CRD) was used to evaluate all quality parameters, except for sensory evaluations, which were assessed using a randomized complete block design. Statistical analyses of mean differences and variations were conducted using ANOVA followed by Tukey's test for multiple comparisons.

## 3 Results and discussion

### 3.1 Nutrition content

The nutritional characteristics of the dried seasoning made from Liang leaves are presented in Table 1. Freshly produced dried seasoning exhibited high levels of protein, fiber, vitamin B_2_, and calcium, making it a good source of vitamin A and iron [26]. According to the Ministry of Public Health [26], the recommended daily dietary intake of protein, dietary fiber, vitamin A, vitamin B_2_, calcium, and iron is 50 g, 25 g, 4.8 mg, 1.7 mg, 800, and 15 mg, respectively. A 28 g serving of the dried seasoning (the recommended portion per meal) provided 14.11 %, 11.45 %, 7.35 %, 8.56 %, 22.48 %, and 6.89 % of the daily nutritional requirements, respectively. After storage for 12 W, a significant increase in the moisture content was observed in samples packaged in nylon/LLDPE (N60 and N90) compared to those stored in laminated aluminum foil (A60 and A90). This increase was attributed to the high moisture permeability of nylon/LLDPE [27]. At high relative humidity of 90 % RH, N90 exhibited the highest moisture content, as this material is highly polar and thus highly sensitive to humidity, leading to rapid moisture permeation. By contrast, the moisture content of samples stored in laminated aluminum foil (A60 and A90) remained relatively stable compared to freshly produced dried seasoning (F), indicating the superior moisture barrier properties of the aluminum foil [27]. Furikake, a traditional dried seasoning, typically has a low moisture content of 2–3% [28]. The moisture content of dried Liang leaf seasoning was slightly higher than that of furikake but remained within a suitable range for use. The protein content of dried seasoning ranged from 25.20 to 32.38 % and exceeded the nutrition claim for a "high protein” food by two to three times (Table 1). This is higher than the protein content reported for furikake made from skipjack and catfish, which typically contains 19.25–23.33 % protein [28]. The protein content of the dried Liang leaf seasoning increased slightly after storage, particularly in samples stored in laminated aluminum foil packaging, likely due to the concentration effect resulting from moisture loss during storage [27]. The dietary fiber content was four times higher than the nutrition claim for a "high fiber” food (Table 1) and significantly exceeded than that of furikake, which is not a substantial source of dietary fiber [28]. This high fiber content could contribute to potential antidiabetic properties, as dietary fiber is known to help regulate blood sugar levels [29]. Throughout the storage period, carbohydrate content showed a notable decrease, whereas fiber and sugar content increased. These variations may be attributed to the hydrolysis of carbohydrates or the formation of new compounds, including xylan and homogalacturonan [30,31]. The highest sugar content was found in A60 after 12 weeks of storage, which may be due to the breakdown of other compounds leading to the release of sugars. Freshly produced dried seasoning had significantly lower fat content than that of furikake, which typically contains 25.81–26.26 % fat [28]. However, the fat content of the seasoning increased significantly during storage, especially in samples stored in laminated aluminum foil at RH 90 %. This increase was likely due to the separation of oil from the matrix after oxidation reaction, which led to the formation of lipid oxidation products [27]. The dried seasoning contained high levels of minerals, particularly calcium (586.27–642.17 mg/100 g) and iron (3.06–3.69 mg/100 g) (Table 1). It points out that this product could be alternative of those important minerals when compare to whole milk containing a calcium content of 114.3 mg/100 g and an iron content of 0.028 mg/100 g [32]. The vitamin A content in dried seasoning met the nutrition claim for a “high vitamin A″ food, though both vitamin A and B_2_ contents decreased after 12 weeks. The degradation of vitamin A was due to exposure to light, high temperature, oxygen, pro-oxidant compounds, and moisture, which can oxidize the unsaturated bonds in vitamin A [33]. Similarly, vitamin B_2_ degradation can occur under light, high temperature, and moist conditions [34]. Overall, the dried Liang leaf seasoning demonstrated a more nutrient-dense profile than furikake, particularly in terms of protein, dietary fiber, and mineral content. Storage conditions and packaging materials significantly influenced the nutritional composition, with laminated aluminum foil proving to be more effective in preserving nutritional quality [27].

**Table 1:** Nutrition content of freshly produced dried seasoning and those after storage for 12 weeks (per 100g wet basis).

Parameter	Treatments					Nutrition claim per 100 g sample (high level)
	F	A60	A90	N60	N90	
Moisture content (g)	4.56	4.59	4.47	10.81	14.77	–
Protein (g)	25.20	32.38	32.30	31.70	29.43	10
Carbohydrate (g)	52.12	41.49	38.95	37.45	34.24	–
Dietary fiber (g)	10.22	22.3	23.71	21.12	19.78	6
Sugar (g)	9.65	16.62	11.77	12.16	10.36	–
Total fat (g)	2.98	6.63	9.58	6.28	8.58	–
Saturated fatty acid (g)	0.70	1.39	1.83	1.36	1.66	–
Cholesterol (mg)	205.07	194.92	244.68	214.97	237.50	–
Ash content (g)	15.14	14.91	14.70	13.76	12.98	–
Sodium (g)	3.49	3.80	3.89	3.25	3.37	–
Vitamin A (mg)	1.26	0.64	0.63	0.67	0.55	1.44
Vitamin B_1_ (mg)	ND	ND	ND	ND	ND	0.45
Vitamin B_2_ (mg)	0.52	ND	ND	ND	ND	0.51
Calcium (mg)	642.17	615.67	586.27	596.47	600.79	240
Iron (mg)	3.69	3.47	3.57	3.09	3.06	4.5
Total energy (kcal)	336.10	355.15	371.22	333.12	331.90	–
Energy from fat (kcal)	26.82	59.67	86.22	56.52	77.22	–

**Remarks:** F: freshly produced dried seasoning; A60: laminated aluminum foil packaging stored at RH 60 %; A90: laminated aluminum foil packaging stored at RH 90 %; N60: nylon/LLDPE packaging stored at RH 60 %; N90: nylon/LLDPE packaging stored at RH 90 %; ND: not detected; –: not regulated; high level: a nutrition claim that describes the high level of a nutrient contained in the food following the notification of the Ministry of Public Health [26].

### 3.2 Moisture content, a_w_ and moisture content change

**Fig. 2:**
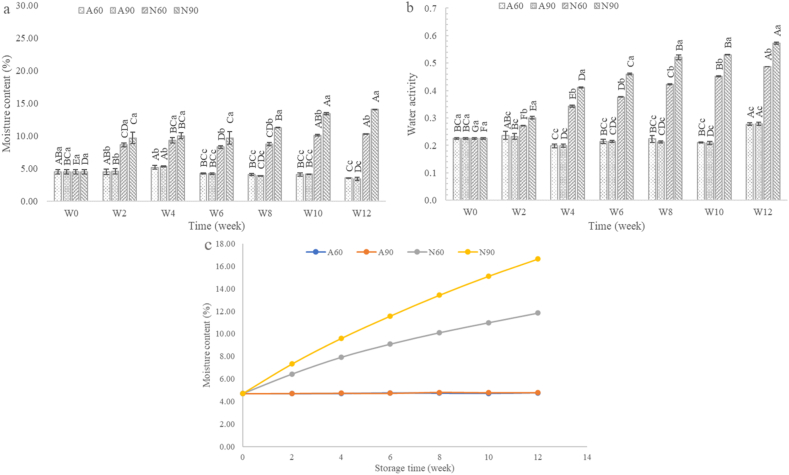
Moisture content (a), water activity (b), and moisture content change (c) of dried seasoning during storage for 12 weeks (n = 3). Different uppercase letters indicate significant differences within the same treatment. Different lowercase letters indicate significant differences between weekly treatments (p < 0.05). A60: laminated aluminum foil packaging stored at RH 60 %; A90: laminated aluminum foil packaging stored at RH 90 %; N60: nylon/LLDPE packaging stored at RH 60 %; N90: nylon/LLDPE packaging stored at RH 90 %; W: week.

Fig. 2(a) shows the significant differences (p < 0.05) in moisture contents of the dried seasoning packaged in laminated aluminum foil and nylon/LLDPE at RH 60 % and RH 90 % during the 12-week storage period. The results indicate that the moisture content varied significantly depending on the relative humidity (RH) and packaging material used. Across all treatments, moisture content increased over time, with the rate of increase being significantly higher in samples stored at RH 90 % than in those stored at RH 60 %. These findings highlight the substantial influence of packaging materials on the moisture content. Specifically, samples packaged in laminated aluminum foil (A60 and A90) consistently exhibited lower moisture content than those packaged in nylon/LLDPE (N60 and N90), which is attributed to the superior moisture barrier properties of the laminated aluminum foil, effectively limiting moisture uptake during storage. The water activity (a_w_) of the dried seasoning, as shown in Fig. 2(b), also displayed significant differences within the same treatment group (p < 0.05), similar to the trends observed for the moisture content. A60 consistently maintained lower water activity throughout the storage period than the other treatments. Notably, at week 12, A60 exhibited the lowest a_w_, emphasizing its superior ability to preserve the quality of dried seasoning by minimizing free water absorption. By contrast, N90 showed the highest a_w_ throughout the storage period, approaching the threshold for microbial growth (a_w_ = 0.6) [35,36] by the end of the storage period. This observation highlights the limitations of nylon/LLDPE in moist environments, where a higher permeability leads to increased free water absorption and elevated a_w_ levels. Additionally, the a_w_ of N90 increased nearly threefold by week 12 compared to its initial value, which is in contrast to the slight increase observed in A60 and A90. Although RH 90 % challenged the moisture barrier properties of laminated aluminum foil, it was more effective than nylon/LLDPE. A stability study was conducted to assess the impact of RH at 30 °C and packaging materials on the moisture content of dried seasoning samples. The moisture content, determined using Equation (2) based on the weight gain during storage, is shown in Fig. 2(c). These results indicate that dried seasoning absorbed moisture from the environment because of its hygroscopic characteristics. Moisture absorption occurred more rapidly at RH 90 % than at RH 60 %, due to the higher availability of moisture in more humid conditions. Moisture content is a critical factor influencing product quality and shelf life, as high moisture levels promote microbial growth, leading to product deterioration [37]. Changes in moisture content are influenced by both the storage environment and permeability of the packaging material [38].

In nylon/LLDPE packaging, dried seasoning reached a critical moisture content of 14.08 % within 10 weeks when stored at RH 90 %. By contrast, dried seasoning packaged in laminated aluminum foil (A60 and A90) exhibited a markedly slower rate of moisture uptake, with the moisture content rising slightly to approximately 5 % after 12 weeks of storage. These findings underscore the importance of selecting appropriate packaging materials to control the moisture uptake. Laminated aluminum foil (A60 and A90) proved to be significantly more effective in limiting moisture absorption than nylon/LLDPE (N60 and N90), due to its lower water vapor permeability coefficient (P). Consequently, using laminated aluminum foil for packaging, particularly under lower RH conditions, is more effective in maintaining a low moisture content and extending the shelf life of dried seasoning.

### 3.3 Water vapor transmission rate (WVTR) and permeability coefficient (P)

The moisture content of dried seasoning is critical for preserving its flavor, texture, and shelf life, making the selection of appropriate packaging materials essential. Laminated aluminum foil, with a thickness of 93.0 μm, exhibited superior water vapor barrier properties compared to nylon/LLDPE with a thickness of 77.6 μm. Specifically, the laminated aluminum foil demonstrated a significantly lower water vapor transmission rate (WVTR) of 3.22 × 10⁻⁶ g/(day × cm^2^) at 25 °C and RH 50 % and a permeability coefficient (P) of 2.52 × 10⁻⁵ g × μm/(day × cm^2^ × mmHg). By contrast, nylon/LLDPE exhibited a WVTR of 7.39 × 10⁻⁵ g/(day × cm^2^) at 25 °C and RH 50 % and a P of 4.83 × 10⁻⁴ g × μm/(day × cm^2^ × mmHg). These findings suggest that laminated aluminum foil is a more effective moisture barrier, which is crucial for maintaining the desired moisture content of dried seasoning over time, particularly in high-humidity environments. The WVTR indicates the amount of water vapor that can permeate through a unit area of the packaging per day. Lower WVTR values indicate better barrier properties of packaging, which are essential for preserving product quality by minimizing moisture ingress. The permeability coefficient (P) measures the rate at which water vapor permeates through a material per unit thickness and per unit pressure gradient across the material. This parameter is fundamental for understanding the intrinsic permeability of a material to water vapor [39]. Based on these permeability coefficients, laminated aluminum foil and nylon/LLDPE can be classified as very high- and high-water barriers, respectively [40]. The superior barrier properties of laminated aluminum foil contribute to enhanced product quality preservation and extended shelf life by reducing the adverse effects of moisture ingress.

### 3.4 Shelf life estimation of dried seasoning

Dried seasoning, a moisture-sensitive food product, often uses moisture content as a primary indicator of shelf life, because of the significant impact of moisture gain or loss on the quality characteristics of the product. In this study, the shelf life was estimated by assuming a constant partial pressure difference across the package wall [41] with a product of 4 g and initial and critical moisture contents of 4.70 % and 14.08 %, respectively, in the pouch. Storage at 30 °C and RH 60 % yielded the longest shelf life, whereas storage at 30 °C and RH 90 % resulted in the shortest shelf life. These variations in shelf life were closely associated with fluctuations in moisture content. The selection of packaging material substantially influenced the shelf life of dried seasoning. The laminated aluminum foil (A60) provided the longest shelf life, preserving acceptable quality up to 684 days. However, when the RH of storage was increased to 90 % (A90), the shelf life was reduced to 275 days, representing a 60 % decrease in shelf life. By contrast, nylon/LLDPE packaging exhibited significantly poor performance owing to inferior moisture barrier properties, with samples lasting only 30 days at 60 % RH (N60) and only 12 days at 90 % RH (N90). These findings suggest that storing dried seasoning in conditions where relative humidity (RH) levels do not exceed 60 %, combined with the use of laminated aluminum foil packaging, effectively prevents moisture permeation from the surrounding environment. This combination of storage conditions and packaging materials resulted in a shelf life of up to 23 months for the dried seasoning product. While moisture content was the primary indicator of shelf life in this, other factors, such as oxidation and microbial stability, also contributed to overall product stability. Thus, the shelf life estimation provided here served as an approximation, assuming constant storage conditions at 30 °C and RH 60 % or RH 90 %. Further studies incorporating additional parameters would yield a more comprehensive shelf life model. These findings underscore the importance of both storage conditions and packaging materials to maintain the quality and extend the shelf life of moisture-sensitive food products such as dried seasoning.

### 3.5 pH value, Brix value and color

**Fig. 3:**
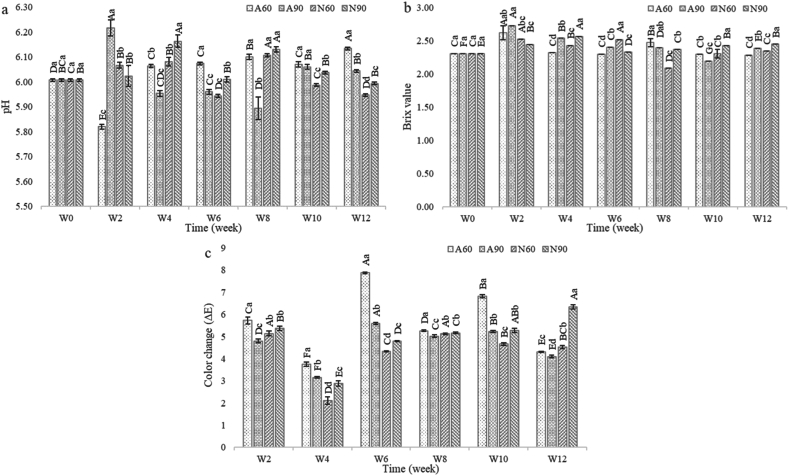
pH (a), Brix (b) and color (c) values of dried seasoning during storage for 12 weeks (n = 3). Different uppercase letters indicate significant differences within the same treatment. Different lowercase letters indicate significant differences between weekly treatments (p < 0.05). A60: laminated aluminum foil packaging stored at RH 60 %; A90: laminated aluminum foil packaging stored at RH 90 %; N60: nylon/LLDPE packaging stored at RH 60 %; N90: nylon/LLDPE packaging stored at RH 90 %; W: week. (For interpretation of the references to color in this figure legend, the reader is referred to the Web version of this article.)

An important observation was the fluctuation in pH, Brix, and color values between fresh produce (weeks 0) and week 2 across all treatments, followed by a general trend of either decreasing, increasing, or stabilizing over the subsequent period. This initial fluctuation may be attributed to moisture gain, which is likely followed by complex reactions and gradual equilibration process. The pH of the dried seasoning was mildly acidic and fluctuated within a narrow range during the 12 weeks of storage (Fig. 3(a)). Changes in pH typically result from the production or degradation of acidic and basic compounds or compounds with buffering capacities [42]. For laminated aluminum foil, samples at RH 60 % (A60) maintained more stable pH levels than those at RH 90 % (A90). A60 exhibited fewer fluctuations. By contrast, dried seasoning packaged in nylon/LLDPE showed higher fluctuations in pH. Samples stored at RH 60 % (N60) generally had lower pH values than those at RH 90 % (N90). This is likely due to the hydrophilic properties of nylon; at higher humidity (90 % RH), nylon likely absorbed more moisture, which could have affected its barrier properties and contributed to the deterioration of dried seasoning. Microorganisms present in the environment can also consume compounds and excrete substances, leading to changes in the pH [43]. The pH changes induced by one species of microorganism can either enhance or inhibit the growth of other microorganisms in the environment [43]. Therefore, fluctuations in pH over storage time are complex and likely caused by interactions between moisture changes, packaging barriers, and the dominant microflora and their metabolic products.

The Brix values of dried seasoning over the 12 W storage period, representing soluble solid content, were low and remained relatively constant at approximately 2.5, with no significant differences among treatments (Fig. 3(b)). However, as the storage time progressed, distinct patterns emerged based on the packaging materials and RH. For the laminated aluminum foil (A60 and A90), those samples stored at both 60 % and 90 % RH maintained relatively stable Brix values throughout the storage period. There were no significant differences between A60 and A90, suggesting that the laminated aluminum foil effectively protected the Brix levels of dried seasoning, regardless of the external humidity level. By contrast, those in nylon/LLDPE (N60 and N90) exhibited more variability over time. Samples stored at RH 60 % (N60) generally maintained lower Brix values than those stored at 90 % RH (N90) did. This trend aligns with the moisture-sensitive nature of nylon, where higher humidity likely led to increased moisture absorption, potentially diluting the soluble solid content and resulting in lower Brix values for N90.

The color of the dried seasoning is primarily influenced by the leaves, spices, and other ingredients used in the formulation. As shown in Fig. 3(c), the total color difference (ΔE) between the dried seasoning and the freshly produced sample (week 0) increased significantly during the first two weeks of storage for all treatments. ΔE values within this range (2–8) indicate color differences that are perceptible to the human eye [44]. Although color continued to change throughout the storage period, no consistent effect of packaging or RH on color change was observed. This lack of consistency can be attributed to the inherent complexity of the dried seasoning. The wide range of ingredients and their varying ratios make it challenging to maintain consistent product coloration and prevent changes during the storage period. Additionally, factors such as the natural pigments in the ingredients, their interactions, and environmental conditions during storage further contribute to the observed color fluctuations.

### 3.6 TPC, TFC and antioxidant activity (DPPH, ABTS and FRAP)

**Fig. 4:**
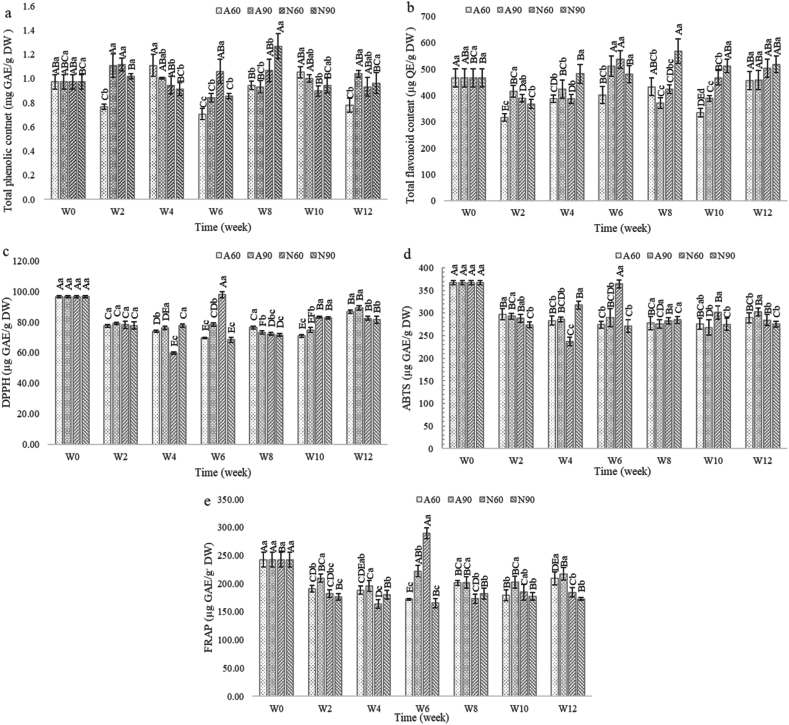
TPC (a), TFC (b), DPPH (c), ABTS (d) and FRAP (e) of dried seasoning during storage for 12 weeks (n = 3). Different uppercase letters indicate significant differences within the same treatment. Different lowercase letters indicate significant differences between weekly treatments (p < 0.05). A60: laminated aluminum foil packaging stored at RH 60 %; A90: laminated aluminum foil packaging stored at RH 90 %; N60: nylon/LLDPE packaging stored at RH 60 %; N90: nylon/LLDPE packaging stored at RH 90 %; W: week.

Despite minor fluctuations, the total phenolic content (TPC) levels remained relatively stable across all treatments, averaging between 0.99 and 1.0 mg GAE/g DW throughout the study (Fig. 4(a)). This consistency suggests a strong retention of phenolic compounds in the seasoning, regardless of the packaging material or relative humidity conditions. This finding aligned with Moraga et al. [45], who reported that the TPC in grapefruit powder remained constant after three months of storage at various RH levels (11%–68 %). By contrast, the total flavonoid content (TFC) exhibited significant fluctuations across all treatments, characterized by irregular increases and decreases throughout the storage period (Fig. 4(b)). These fluctuations were statistically significant (p < 0.05), indicating that storage time and conditions had an impact on both the TFC and TPC levels. The observed variations in TFC and TPC highlight the complex characteristics of phytochemical preservation in dried seasoning. During storage, oxidation of antioxidant compounds may occur, leading to a decrease in antioxidant activity [46]. However, in certain cases, the oxidation of phenolic compounds can result in the formation of new antioxidant polymers, which initially enhance antioxidative activity before it declines as the polymer chains lengthen [46]. While laminated aluminum foil showed a slight advantage in maintaining high levels of both TPC and TFC, the inconsistent patterns observed across treatments over time emphasize the importance of careful interpretation in storage studies. The antioxidant activities of the dried seasoning, as measured by DPPH, ABTS, and FRAP assays, exhibits complex dynamics over the 12-week storage period under varying packaging and humidity conditions (Fig. 4(c), (d), and 4(e)). Antioxidant activity is attributed to the change in vitamin C content, which is a water-soluble antioxidant produced in plants [47]. Vitamin C levels typically decrease with increasing temperature, storage time, and moisture content [48]. The DPPH assay demonstrated relatively stable free radical scavenging activity, with a narrow range fluctuation (78.8–81.46 μg GAE/g DW), suggesting the resilience of certain antioxidant compounds during storage. This stability is consistent with the findings of Moraga et al. [45] who reported that the antioxidant activity of grapefruit powder in a glassy state remained constant. In this state, the limited molecular movement of total phenols, flavonoids, vitamin C, and organic acids contributed to their preservation, whereas, in the rubbery state, bioactive compounds deteriorated between 3 and 6 months of storage, with this deterioration increasing with higher RH or moisture reabsorption [45]. Conversely, the ABTS assay exhibited a higher range of values (294.81–303.48 μg GAE/g DW), indicating its potential sensitivity to a broader spectrum of antioxidants in the seasoning. The FRAP assay displayed the widest range (172.87–217.33 μg GAE/g DW) and the most pronounced fluctuations, suggesting that the reducing agents in the seasoning are more susceptible to storage conditions. Among the assays conducted, the ABTS assay exhibited the highest antioxidant activity, followed by the FRAP assay, and the DPPH assay showed the lowest activity. The high antioxidant value in the ABTS assay indicates that the major compounds in the product were polar, providing hydrogen or electron transfer capability to radicals and metal ion reduction (Fe^3+^ to Fe^2+^) as measured by the FRAP assay. Moreover, across all assays, laminated aluminum foil packaging (A60 and A90) consistently outperformed nylon/LLDPE (N60 and N90), with lower relative humidity (60 %) generally yielding better antioxidant preservation. Table 2 shows the observed variations in the percentage retention of total phenolic content (TPC), total flavonoid content (TFC), and antioxidant activities (DPPH, ABTS, and FRAP), reflecting the inherent biological variability of natural raw materials. Notably, some measurements exceeded 100 % of the initial values, particularly for TPC and TFC. This phenomenon may be attributed to the natural variation in plant-based materials and potential changes in the matrix during storage, which could enhance extraction efficiency or compound availability. These increases may result from the breakdown of complex phenolic compounds into simpler forms or the release of bound phenolics during storage. The antioxidant activity assays (DPPH, ABTS, and FRAP) also exhibited fluctuations, with some values exceeding 100 %. This variability could be due to the formation of new antioxidant compounds during storage or synergistic effects among existing antioxidants [46]. Importantly, all conditions maintained relatively stable levels of bioactive compounds and antioxidant activities throughout the 12 weeks, with most values remaining at least 70 % of the initial measurements. This suggests that both packaging materials are effective in preserving the antioxidant properties of dried seasonings in the short term. Future studies should consider longer storage periods and more frequent sampling to better characterize the complex dynamics of these natural products during storage.

**Table 2:** Proportion of TPC, TFC, and antioxidant activity in dried seasoning during storage for 12 weeks compared to freshly produced dried seasoning at week 0 (as 100 %) (n = 3).

Condition/Time	A60	A90	N60	N90
TPC				
W2	78.68 ± 2.22^Cb^	113.55 ± 9.97^Aa^	114.63 ± 5.53^Aa^	104.57 ± 2.28^Ba^
W4	113.92 ± 9.40^Aa^	103.05 ± 0.50^ABab^	97.03 ± 7.60^ABb^	93.83 ± 5.44^BCb^
W6	72.51 ± 4.66^Cc^	86.52 ± 3.81^Cb^	108.79 ± 10.18^ABa^	87.78 ± 2.40^Cb^
W8	96.86 ± 3.56^Bb^	95.74 ± 5.05^BCb^	109.78 ± 9.52^ABb^	130.16 ± 10.65^Aa^
W10	108.22 ± 4.73^ABa^	102.80 ± 3.08^ABab^	92.18 ± 4.49^Bb^	96.85 ± 6.25^BCab^
W12	79.93 ± 5.90^Cb^	106.75 ± 2.82^ABa^	95.72 ± 8.06^ABab^	98.79 ± 8.85^BCa^
TFC				
W2	67.91 ± 3.02^Cc^	89.33 ± 4.79^BCa^	83.50 ± 3.18^Dab^	78.89 ± 3.58^Cb^
W4	83.13 ± 2.89^Bb^	90.93 ± 7.45^BCb^	82.92 ± 3.74^Db^	103.40 ± 7.64^Ba^
W6	86.17 ± 6.77^Bb^	109.63 ± 8.14^Aa^	115.10 ± 7.03^Aa^	103.09 ± 6.85^Ba^
W8	92.90 ± 7.13^ABb^	79.71 ± 4.33^Cc^	91.02 ± 3.37^CDbc^	121.80 ± 10.08^Aa^
W10	71.79 ± 3.61^Cd^	83.39 ± 2.25^Cc^	100.29 ± 2.25^BCb^	109.86 ± 5.42^ABa^
W12	97.77 ± 7.41^Aa^	98.29 ± 7.94^ABa^	107.87 ± 7.55^ABa^	111.00 ± 6.73^ABa^
DPPH				
W2	80.29 ± 0.83^Ba^	81.98 ± 0.77^Ba^	81.02 ± 2.35^Ca^	80.52 ± 2.45^Ba^
W4	76.51 ± 0.83^Cb^	78.92 ± 1.14^CDa^	61.96 ± 0.63^Ec^	80.44 ± 1.27^Ba^
W6	72.19 ± 0.47^Dc^	81.24 ± 1.13^BCb^	101.38 ± 2.03^Aa^	70.80 ± 1.55^Cc^
W8	79.22 ± 0.99^Ba^	75.90 ± 1.02^Eb^	74.93 ± 0.82^Dbc^	74.09 ± 0.89^Cd^
W10	73.49 ± 0.97^Dc^	77.55 ± 1.81^DEb^	86.21 ± 0.53^Ba^	85.65 ± 0.88^Aa^
W12	89.99 ± 1.11^Aa^	92.47 ± 1.62^Aa^	85.45 ± 1.21^Bbc^	84.45 ± 2.48^Ab^
ABTS				
W2	81.00 ± 3.37^Aa^	79.85 ± 1.74^ABa^	78.50 ± 2.25^Cab^	74.60 ± 1.68^Bb^
W4	77.17 ± 2.63^ABb^	77.87 ± 1.45^ABCb^	64.57 ± 2.53^Bc^	86.51 ± 2.32^Aa^
W6	74.74 ± 2.13^Bb^	79.03 ± 5.51^ABCb^	99.31 ± 2.39^Aa^	73.76 ± 3.87^Bb^
W8	75.63 ± 3.98^ABa^	75.10 ± 2.59^BCa^	77.06 ± 2.02^Ba^	77.58 ± 2.06^Ba^
W10	75.22 ± 3.39^ABab^	72.96 ± 4.61^Cb^	82.07 ± 3.97^Ba^	74.88 ± 3.72^Bb^
W12	78.90 ± 3.09^ABab^	82.39 ± 2.39^Aa^	77.40 ± 3.34^Bb^	75.05 ± 1.68^Bb^
FRAP				
W2	78.71 ± 2.44^BCb^	86.39 ± 2.94^ABCa^	75.12 ± 2.95^Bbc^	72.78 ± 2.32^Abc^
W4	77.66 ± 3.03^BCab^	80.59 ± 4.00^Ca^	67.47 ± 3.16^Cc^	74.28 ± 3.26^Ab^
W6	71.04 ± 0.57^Dc^	91.64 ± 4.11^Ab^	119.39 ± 3.89^Aa^	68.21 ± 3.37^Bc^
W8	82.94 ± 1.85^ABa^	83.26 ± 4.24^BCa^	71.39 ± 3.40^BCb^	75.05 ± 4.12^Ab^
W10	74.04 ± 4.05^CDb^	83.36 ± 4.40^BCa^	76.24 ± 5.78^Bab^	73.14 ± 2.81^Bb^
W12	86.14 ± 4.77^Aa^	89.58 ± 4.63^ABa^	76.05 ± 3.53^Bb^	71.25 ± 1.10^ABb^

**Remarks:** Different uppercase letters indicate significant differences within the same treatment. Different lowercase letters indicate significant differences between weekly treatments (W) (p < 0.05). A60: laminated aluminum foil packaging stored at RH 60 %; A90: laminated aluminum foil packaging stored at RH 90 %; N60: nylon/LLDPE packaging stored at RH 60 %; N90: nylon/LLDPE packaging stored at RH 90 %; W: week.

### 3.7 Microbiological quality

Initially, the freshly produced seasoning exhibited relatively low microbial counts with total viable count (TVC) of 2.66 log CFU/g and yeast and mold (YM) of 1.90 log CFU/g. However, prolonged storage revealed potential risks concerning TVC and YM quality. At week 6, both TVC and YM counts increased across both types of packaging and under both humidity conditions, reaching approximately 3 log CFU/g for TVC and 2 log CFU/g for YM. This increase highlights the potential for microbial growth during storage. By week 12, a noticeable reduction in microbial counts was observed in laminated aluminum foil at 60 % relative humidity (RH), with TVC and YM levels dropping to 2.04 and 1.34 log CFU/g, respectively. These results suggest that laminated aluminum foil was more effective in controlling microbial growth over a longer period, particularly at lower humidity levels, and that relative humidity played a significant role in the microbial quality of packaged food products. Our findings indicated that lower humidity (RH 60 %) generally supported lower microbial growth than RH 90 % across both types of packaging. By week 12, microbial counts in laminated aluminum foil and nylon/LLDPE were lower at RH 60 % than at RH 90 %, where TVC and YM exceeded 2.4 log CFU/g and 1.7 log CFU/g, respectively. This trend was consistent with the understanding that higher moisture levels promote microbial proliferation. High TVC and YM in this study were critical indicators of microbial activity, potentially leading to spoilage and reduced product quality. This observation aligned with Agalloco et al. [49] and Siripongvutikorn et al. [50] who reported similar microbiological concerns regarding Thai furikake. Although pathogenic microorganisms such as *E. coli* were not detected, the observed microbial load increased over storage duration. Thus, there is still a need for process improvement to reduce microbial contamination risks at all stages, from raw material handling to preparation and drying. Further study should explore the integration of active packaging technologies, such as oxygen scavengers or antimicrobial agents, to enhance microbial stability and safety of the product. In addition, a broader range of pathogens and spoilage organisms should be tested to provide a more comprehensive safety profile.

### 3.8 Sensory evaluation

**Fig. 5:**
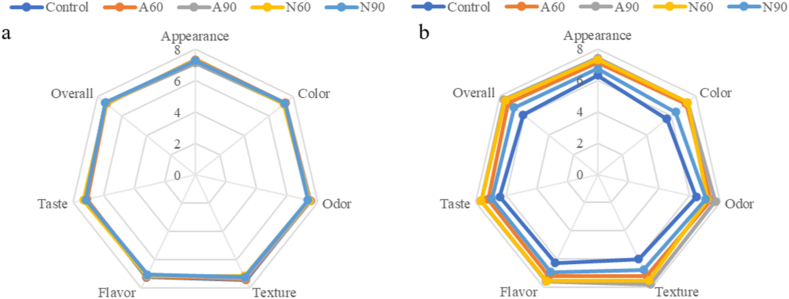
Web diagram of the dried seasoning sensory scores after storage for 2 weeks (a), and 12 weeks (b) compared to freshly produced dried seasoning (control: 0 week) (n = 50).

The sensory scores presented in Table 3 for appearance, color, odor, texture, flavor, taste, and overall acceptability were generally higher for samples stored in laminated aluminum foil (A60 and A90) than for those stored in nylon/LLDPE (N60 and N90) after 12 weeks. This suggests that laminated aluminum foil provided better protection against quality deterioration during storage than nylon/LLDPE. Additionally, samples stored at RH 60 % (A60 and N60) exhibited higher sensory scores than those stored at RH 90 % (A90 and N90) after 12 weeks, particularly those stored in nylon/LLDPE. The lowest sensory score was observed for N90, which had the highest water activity (a_w_). Increased moisture absorption and high a_w_ are critical factors that accelerate changes in physical, chemical, and microbial quality, leading to lower sensory scores [51]. Although the a_w_ of N90 at 12 weeks did not exceed 0.6, which is typically required for microbial growth, enzymatic reactions, browning, and oxidation reactions still occur [28]. At W2, the sensory scores of all samples, including freshly produced dried seasoning (control), were similar (Fig. 5(a)). However, by week 12, distinct differences were observed between the packaging treatments and control (Fig. 5(b)). Notably, the sensory scores of freshly produced dried seasoning stored at freezing temperatures were slightly lower than those of the stored samples, suggesting that a product may require a period of stabilization to achieve ingredient or component equilibrium, similar to the aging effect observed in some food products [52].

**Table 3:** Sensory scores of dried seasonings after storage for 2 and 12 weeks (n = 50).

Characteristic	Condition/Time	F	A60	A90	N60	N90
Appearance	W2	7.26 ± 1.35^a^	7.32 ± 0.98^a^	7.12 ± 1.32^a^	7.28 ± 1.11^a^	7.26 ± 1.03^a^
	W12	6.32 ± 1.42^c^	7.12 ± 1.02^ab^	7.42 ± 1.05^a^	7.30 ± 0.89^ab^	6.72 ± 1.50^bc^
Color	W2	7.24 ± 1.29^a^	7.34 ± 0.98^a^	7.24 ± 1.13^a^	7.22 ± 1.00^a^	7.30 ± 0.93^a^
	W12	5.66 ± 1.49^c^	7.22 ± 0.95^a^	7.34 ± 0.92^a^	7.34 ± 0.82^a^	6.36 ± 1.31^b^
Odor	W2	7.48 ± 1.36^a^	7.54 ± 1.03^a^	7.50 ± 1.04^a^	7.44 ± 0.99^a^	7.36 ± 1.24^a^
	W12	6.46 ± 1.30^c^	7.40 ± 0.88^ab^	7.70 ± 0.84^a^	7.18 ± 1.02^ab^	7.08 ± 0.94^b^
Texture	W2	7.44 ± 1.36^a^	7.40 ± 0.99^a^	7.34 ± 1.04^a^	7.18 ± 1.26^a^	7.26 ± 1.23^a^
	W12	6.00 ± 1.43^c^	7.20 ± 1.05^ab^	7.78 ± 0.82^a^	7.52 ± 1.11^a^	6.76 ± 1.35^b^
Flavor	W2	7.26 ± 1.59^a^	7.20 ± 1.09^a^	7.10 ± 1.22^a^	7.16 ± 1.15^a^	7.10 ± 1.27^a^
	W12	6.26 ± 1.34^c^	7.18 ± 1.14^ab^	7.58 ± 0.84^a^	7.54 ± 0.91^a^	6.92 ± 1.08^b^
Taste	W2	7.20 ± 1.60^a^	7.10 ± 1.13^a^	7.20 ± 1.23^a^	7.32 ± 1.35^a^	7.18 ± 1.26^a^
	W12	6.40 ± 1.28^c^	7.18 ± 1.35^ab^	7.64 ± 0.88^a^	7.62 ± 0.99^a^	6.92 ± 1.12^bc^
Overall acceptability	W2	7.32 ± 1.41^a^	7.26 ± 1.05^a^	7.32 ± 1.00^a^	7.30 ± 1.13^a^	7.36 ± 1.10^a^
	W12	6.08 ± 1.28^c^	7.30 ± 0.99^a^	7.64 ± 0.75^a^	7.56 ± 0.93^a^	6.84 ± 0.89^b^
Acceptance	W2	91.84	89.8	100	87.76	87.76
	W12	97.96	100	97.96	87.76	69.39

**Remarks:** Different lowercase letters indicate significant differences between treatments within the same period (p < 0.05). F: freshly produced dried seasoning; A60: laminated aluminum foil packaging stored at RH 60 %; A90: laminated aluminum foil packaging stored at RH 90 %; N60: nylon/LLDPE packaging stored at RH 60 %; N90: nylon/LLDPE packaging stored at RH 90 %; W: week.

## 4 Conclusions

A novel shelf-stable Liang leaf seasoning was developed and characterized by high levels of protein, fiber, vitamins, minerals, and antioxidants. The quality and shelf life of a product were significantly influenced by the packaging materials and storage conditions. The dried Liang leaf seasoning was a good source of daily protein (14.11 %) and calcium (22.48 %) intake. The laminated aluminum foil demonstrated superior moisture barrier properties, offering an estimated shelf life of up to 684 days at RH 60 %. The total phenolic content (TPC) remained stable throughout storage, while the total flavonoid content (TFC) fluctuated significantly, reflecting the complexity of phytochemical preservation. DPPH, ABTS, and FRAP assays, responded and reflected to antioxidant activity in different ways, with DPPH showing stability, ABTS indicating a broader sensitivity to antioxidants, and FRAP exhibiting the most fluctuation. Moreover, laminated aluminum foil packaging at RH 60 %, proved to be more effective in controlling microbial growth and received the highest sensory scores. These findings suggest that laminated aluminum foil, combined with lower relative humidity, was highly effective in extending the shelf life and preserving the nutritional quality of dried seasoning. Furthermore, this study highlights the potential of Liang leaves as a sustainable seasoning ingredient with high nutrition value and health benefits. Future research should investigate longer storage durations and additional quality attributes such as antioxidant activity, texture and microbial stability for a more robust shelf life prediction, as well as the incorporation of active packaging to further enhance the stability and safety of the product.

## CRediT authorship contribution statement

**Worapong Usawakesmanee:** Writing – review & editing, Validation, Supervision, Resources, Project administration, Funding acquisition, Conceptualization. **Supachai Pisuchpen:** Writing – review & editing, Validation, Supervision, Project administration, Methodology, Investigation, Formal analysis, Data curation. **Sunisa Siripongvutikorn:** Writing – review & editing, Validation, Supervision, Resources, Project administration, Data curation. **Nicha Khatcharin:** Investigation, Formal analysis, Data curation. **Chanonkarn Rujirapong:** Writing – original draft, Investigation, Formal analysis, Data curation.

## Ethic statement

This study was conducted in accordance with the ethical guidelines outlined by the PSU Human Research Ethics Committee. Ethical approval for the sensory evaluation experiments was obtained from the PSU Human Research Ethics Committee, reference number 2023-005-1-1. All participants were fully informed about the nature of the study, including any potential risks and benefits, and provided their informed written consent prior to their participation.

## Data availability statement

The data that support the findings of this study are available from the corresponding author upon reasonable request.

## Funding

This research received financial support through a grant (AGR6505112M/AGR6505112d) jointly provided by the National Science, Research and Innovation Fund (NSRF) and 10.13039/501100004508Prince of Songkla University.

## Declaration of competing interest

The authors declare that they have no known competing financial interests or personal relationships that could have appeared to influence the work reported in this paper.
